# Balloon Cell Urethral Melanoma: Differential Diagnosis and Management

**DOI:** 10.1155/2015/919584

**Published:** 2015-07-14

**Authors:** M. McComiskey, C. Iavazzo, M. Datta, R. Slade, B. Winter-Roach, G. Lambe, V. K. Sangar, M. Smith

**Affiliations:** ^1^Gynaecological Oncology Department, Christie Hospital, Manchester M20 4BX, UK; ^2^Plastic Surgery Department, Christie Hospital, Manchester M20 4BX, UK; ^3^Urology Department, Christie Hospital, Manchester M20 4BX, UK

## Abstract

*Introduction.* Primary malignant melanoma of the urethra is a rare tumour (0.2% of all melanomas) that most commonly affects the meatus and distal urethra and is three times more common in women than men.* Case.* A 76-year-old lady presented with vaginal pain and discharge. On examination, a 4 cm mass was noted in the vagina and biopsy confirmed melanoma of a balloon type. Preoperative CT showed no distant metastases and an MRI scan of the pelvis demonstrated no associated lymphadenopathy. She underwent anterior exenterative surgery and vaginectomy also. Histology confirmed a urethral nodular malignant melanoma.* Discussion.* First-line treatment of melanoma is often surgical. Adjuvant treatment including chemotherapy, radiotherapy, or immunotherapy has also been reported. Even with aggressive management, malignant melanoma of the urogenital tract generally has a poor prognosis. Recurrence rates are high and the mean period between diagnosis and recurrence is 12.5 months. A 5-year survival rate of less than 20% has been reported in balloon cell melanomas along with nearly 20% developing local recurrence.* Conclusion.* To the best of our knowledge, this case is the first report of balloon cell melanoma arising in the urethra. The presentation and surgical management has been described and a literature review provided.

## 1. Introduction

Primary malignant melanoma of the urethra is a rare tumour (0.2% of all melanomas) that most commonly affects the meatus and distal urethra and is three times more common in women than men [1–6]. Clinical examination usually reveals a haematoma-like tumour at the external urethral meatus. It is mainly a disease of elderly women with an average age at presentation of 64 years and is probably related to UV light exposure [3].

Staging includes extension, depth, necrosis and lymph node, and vascular invasion. In addition to the TNM classification system, tumour extent is staged by using Clark Level, Breslow Index, and Chung Level [3]. General prognosis appears to be poor, with the T-stage as a basis of depth invasion, mucosal location, nodular growth, pulmonary metastases, local recurrence, and systemic recurrence being the main prognostic factors [1, 5].

Differential diagnosis includes transitional cell carcinoma, sarcomatoid carcinoma, and sarcomas. One of the rarest subtypes of urethral melanomas is the balloon cell type which is a rare histologic variant of cutaneous malignant melanoma with exceptional reports of occurrences at noncutaneous sites such as in brain, choroid, and anus (Table 1) [7–9]. It is the rarest histological type of primary cutaneous melanoma and is composed of large, polyhedral, foamy cells with abundant cytoplasmic vacuoles [10, 11].

This report describes the presentation and management of a patient with balloon cell urethral melanoma. A review of the relevant literature is also presented.

## 2. Case

Ms CJ was a 76-year-old lady who presented with vaginal pain and discharge. She had a past medical history of asthma, hypertension, hyperlipidaemia, type II diabetes mellitus, and congenital right hydronephrosis (due to a congenital vascular abnormality, treated with a ureteric stent). She had a left salpingo-oophorectomy for a nonmalignant lesion of the ovary.

On examination she was noted to have a 4 cm mass in the vagina and biopsy of this confirmed melanoma of a balloon type. Preoperatively CT scan did not show any evidence of distant metastases and an MRI scan of the pelvis demonstrated the lesion, but no associated lymphadenopathy (T4N0M0). She was discussed at the regional tumour-board meeting and considered for neoadjuvant treatment; however no appropriate systemic therapy was available. She therefore underwent extensive surgery consisting of an anterior exenteration in which her bladder, uterus, and remaining right ovary were removed. Intraoperative concerns regarding attaining clear margins lead to the decision to perform vaginectomy also. Tumour was resected completely and Ms CJ made a good recovery and experienced no perioperative complications. Histology confirmed a urethral nodular malignant melanoma. Further discussion was performed at the melanoma tumour-board meeting with the decision of close follow-up. Gynaecological oncology clinic review at 3 weeks and 12 weeks postoperatively revealed no complications.

Macroscopic histopathological examination revealed a pelvic exenteration specimen measuring 140 × 135 × 62 mm. It comprised vagina measuring 66 × 49 × 25 mm which contained a pigmented and nodular tumour measuring 28 × 25 × 21 mm which lay 20 mm from the distal resection margin. The tumour was situated on the anterior wall of the vagina. The specimen also contained a bladder measuring 40 × 38 × 36 mm, the mucosa of which appeared normal. There was attached urethra measuring up to 30 mm in length which appeared close to the tumour distally.

Microscopic examination showed a tumour arising from the urethra which showed squamous metaplasia. The lesion was nodular and was composed of cytologically atypical cells with pigmentation in areas. The cells were pleomorphic and hyperchromatic and some cells showed prominent nucleoli. There were epithelioid areas, along with areas showing cleared cytoplasm and elsewhere the tumour displayed a spindle cell architecture. There were occasional atypical multinucleated cells. Overall the features were of a nodular malignant melanoma. The lesion measured 22 mm in thickness and there was evidence of ulceration within the urethral epithelium. Six mitotic figures were identified per 10 high power fields. There were no lymph node invasion identified and no lymphovascular, perineural invasion or microsatellites. The tumour abutted the circumferential margin but did not infiltrate into it and lay 19 mm from the distal resection margin.

## 3. Discussion

Malignant urethral melanomas usually arise from the distal part of the urethra with a variety of symptoms including a pigmented urethral mass with variable colour which could be firm, nodular, or ulcerated. Other nonspecific symptoms include perineal pain, dysuria, incontinence, haematuria or local bleeding, and pruritus [3]. A recent review revealed that the first-line of treatment is surgery including procedures such as tumour excision, cystectomy, ureterostomy, bilateral inguinal and pelvic lymph node dissection, total urethrectomy, or pelvic exenteration [1]. Our case was treated with pelvic exenteration. No lymphadenectomy or sentinel-lymph node biopsy was performed based on the fact that no enlarged lymph nodes were found in the MRI. Adjuvant treatment including chemotherapy, radiotherapy, or immunotherapy such as dacarbazine, nimustine, vincristine, and beta-interferon has also been reported [4]. More specifically, the treatment of urethral melanoma is similar to that of cutaneous malignant melanomas because of lack of experience caused by the rarity of the tumour; therefore adjuvant treatment with interferon-alpha or ipilimumab has been recommended [3].

A clinicopathological analysis of urethral melanomas revealed that disease involved the distal urethra, usually the meatus, and was typically polypoid, ranging from 0.8 to 6 cm (mean, 2.6 cm) in maximum diameter [5]. A vertical growth phase was present in all tumours, and a prominent nodular component was present in the majority of them [5]. The depth of invasion ranged from 2 to 17 mm. The tumours were characterised by diffuse, nested, storiform, or mixed growth patterns [5]. The neoplastic cells typically had abundant eosinophilic cytoplasm, large nuclei with prominent nucleoli, and brisk mitotic activity [5]. Melanin pigment was a common finding while the majority of the tumours were confined to the urethra without lymph node metastasis [5].

Regarding balloon cell melanoma, the characteristic cells should be differentiated from foamy macrophages [9]. Usually, they do not contain mucin and glycogen or lipid is variable, while a few demonstrate staining for Masson's Fontana [9]. Immunohistochemically, it shows immunoreactivity (focal or variable) for S100 protein, HMB-45, Melan-A, microphthalmia-transcription-factor, and tyrosinase while it is negative for CK, CD117, SMA, desmin, synaptophysin, chromogranin, and CD68 [9].

Even with aggressive management, malignant melanoma of the urogenital tract generally has a poor prognosis [2]. Recurrence rates are high (71.4%) and could comprise either local recurrences or metastases in the inguinal lymph nodes [1]. The mean period between diagnosis and recurrence is 12.5 months [1]. Robust prognostic data specifically relating to balloon cell melanoma are lacking. Interestingly, the prognostic indicator of degree of balloon cell change is no longer thought to be important. Rather, the tumour thickness is used in modern practice to give some prognostic information [11–13]. A 5-year survival rate of less than 20% has been reported in balloon cell melanomas [10] along with nearly 20% developing local recurrence and more than half of patients deceased at 2–12 years from metastatic disease [11].

## 4. Conclusion

To the best of our knowledge, this case is the first report of balloon cell melanoma arising in the urethra. The presentation and surgical management has been described, along with the difficulties regarding practising evidence-based medicine in this subtype of melanoma. Counselling patients regarding potential outcome is difficult, also due to a relative paucity of data. However surgical treatment may be radical and outlook should be cautious.

## Figures and Tables

**Table 1:** Balloon cell melanoma literature review with focus on treatment/outcome.

Author	Year	Article type	Number of patients	Treatment	Outcome	Comments
Bal et al. [9]	2013	Case report	1 (anal, primary)	Standard	<20% 5-year survival	

Richardson et al. [14]	2012	Case report	1 (brain, secondary)	Chemo and surgery provide best outlook		

Lee et al. [12]	2011	Case report/lit. review	1	Standard		Prognosis of BCMM does not depend on the degree of balloon cell change, tumor size, nuclear atypia, or mitotic activity

Kao et al. [11]	1992	Cohort study	34	Standard	57.5% died at 2–12 years from metastasis. 18.2% developed local recurrences: 15.2% alive with metastatic tumours 21.2% alive without evidence of disease	Prognosis of BCMM usually correlates with the tumour thickness

Peters and Su [15]	1985	Case report/lit. review	1	Standard		Many balloon cell melanomas show only mild cellular atypia and minimal mitotic figures although the tumour has a malignant course.

Fievez [16]	1984	Lit. review	Unknown	Unknown	6.3-year median survival	

Jakobiec et al. [13]	1979	Case series	2 (ciliary body)	Unknown		Abundant number of balloon cells probably comparatively dormant and benign tumours
